# Fine Art and Good Health for the Masses

**DOI:** 10.3201/eid1207.AC1207

**Published:** 2006-07

**Authors:** Polyxeni Potter

**Affiliations:** * Centers for Disease Control and Prevention, Atlanta, Georgia, USA

**Keywords:** Alfons Mucha, le style Mucha, art nouveau, Zodiac, Henry van de Velde ,

**Figure:**
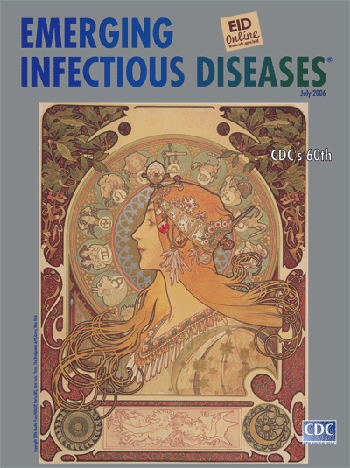
Alfons Mucha (1860–1939). Zodiac (1896). Color lithograph (46 cm × 35 cm). Copyright 2006 Mucha Trust/ADAGP, Paris/ARS, New York. Photo, The Bridgeman Art Library, New York

Art is eternal, Alfons Mucha maintained, so it could never be merely "nouveau" (*1*). Contradicting those who saw him as part of a larger art movement, he insisted that his work was his alone. He followed his own creative impulses, was inspired by Czech folk traditions, and sought the spiritual in art. Yet, his palette so expressed the aesthetics of art nouveau that the movement was dubbed *le style Mucha*.

In cities across Europe and North America, amidst sweeping modernization and mechanization in the 1870s to the 1900s, art nouveau embraced all forms and designs, encompassed diverse styles and media, and remained influential well into the 20th century (*2*). Born of discontent with existing notions and styles, the movement fueled experimentation and reform and shattered the barriers between fine arts (painting, sculpture) and applied arts (ceramics, glassware, furniture, textiles, metalwork). Revolt against convention at times went hand in hand with political revolt against oppressive regimes, as was the case in Mucha's native Moravia, now Czech Republic, then still part of the Austrian and Austro-Hungarian Empire.

Folklore was plentiful in Ivančice, Mucha's hometown near Brno, as were Eastern religious traditions and Slav nationalism, all of which colored his work. Although he reputedly started to draw before he could walk, his talent for music was recognized earlier than his gift for art. As a child and throughout his youth, he sang in the cathedral choir in Brno. Religious images on the walls of the cathedral and other churches awakened his interest in art, particularly drawing. But when he applied for admission to Prague's Academy of Fine Arts, he was rejected: "Find yourself another profession where you'll be more useful" was the academy's recommendation (*3*). He left for Vienna, starting his career as scene painter in the theater. In Munich and then Paris, he received formal art training and worked on magazine and theater designs.

He earned a modest living from illustrations and lithographs and knew Gauguin before his legendary trip to Tahiti, when they briefly shared a studio above the crémerie on Rue de la Grande Chaumière. When they met again 2 years later, "The poor insignificant painter whom Gauguin had known at Madame Charlotte's in 1891 was safely on the way to success…" (*4*). Mucha's career took off when he stepped in at the last minute to design an advertising poster for Sarah Bernhardt, then the most famous actress in Paris. The poster (*1*), created in 2 weeks as an advertisement for Gismonda, became an instant sensation. Its distinctive elongated shape, muted colors, and elaborate decor became an icon and launched a lucrative association between Bernhardt and Mucha. His innovative designs complemented the actress' striking persona.

Some of Mucha's best work (advertising posters, jewelry, theater sets and costumes, book illustrations, carpet and wallpaper designs), on fine paper or fabric, was created at this time. Originals were translated into popular reproductions on matchboxes, postcards, calendars, and home designs, all illustrated with a richness reminiscent of the Byzantine icons he loved and collected. Accessible and dynamic, they became part of the vernacular, their figures harmoniously integrated with the surroundings in complex linear compositions. Curves, spirals, and intricate ornamentation spilled over into architectural and folk designs and dominated graphic art.

"We can't allow a split… ranking one art above the others," wrote artist Henry van de Velde in 1895, articulating a doctrine of art nouveau: art should affect the lives of all people, should enter their homes and influence their furnishings, uniting beauty and utility (*2*). Even mass-produced machine-made objects (stamps, money, lottery tickets, police uniforms) should be guided by sound design. To emphasize the social character of art, such projects as an International Exhibition of Art and Popular Hygiene sought to bring art to public facilities, public houses, and railway stations. The graceful organic shapes of Paris Métro entrances (Hector Guimard, 1867–1942) exemplified this principle (*5*).

Nature, a main source of inspiration, stood for modernity. With publication in 1859 of The Origin of Species and the development of evolutionary theory, progress in culture began to be viewed as analogous to evolution in nature. Rare and exotic plants and animal forms seen under the microscope found themselves in home and other designs as many artists became versed in natural history and biology and published in academic journals of those fields. Undulating, nongeometric "whiplash" curves, hyperbolas and parabolas, and intertwined organic forms dominated everything from jewelry design (René Lalique in Paris) to glassware (Louis Comfort Tiffany in New York).

For Mucha the astonishing success of his popular designs was only a prelude to what he considered his best work, The Slav Epic, a monumental painting inspired by his devotion to the Czech people. He died of pneumonia before the work was finished. Reaction to the painting was mixed, and his fame, particularly among his compatriots, diminished, to be revived again during the 1960s and remain strong to this day.

Zodiac, on this month's cover, shows why Mucha's work was instantly popular. The image exudes comfortable familiarity even as it invites contemplation. The human figure and its surroundings, harmonious and integrated, are evocative of nature. Floral and celestial elements are arranged symmetrically around a portrait, the focus of the intricate composition. Gaze and posture show directness, innate confidence, a sense of self. Filled with energy and movement, she is the dominant star in the celestial sphere. And "flowering" into the complex botanical frame, she captivates with poise and modesty. An exotic gypsy queen, she holds the mysteries of the zodiac, the flow of time, the riddles of nature, the fortunes of the world, deriving her power and magnetism from the symbols surrounding her, in perfect harmony and balance with the content of the universe.

A common motif in Mucha's work, the zodiac alludes to the birth of life and tries to identify and define it, predict its course, and control its outcome. Derived from the artist's faith in the spiritual aspect of art and the power of tradition as source of inspiration, it does what art nouveau sought to do, elevate folk elements to fine art accessible to everyone.

The desire near the end of the 19th century to beautify and advance the world culturally as it was advancing and evolving scientifically is understandable. Social purpose in art, which graces the mundane for the common people, is no different from social purpose in medicine, which improves and extends their lives. Public service for the greater good is like fine art for the masses. Mucha's Zodiac seems a fitting astrologic birthday card, as the Centers for Disease Control and Prevention celebrates 60 years of identifying, describing, explaining, and preventing unknown elements for the benefit of humanity.
